# Bioleaching as an Eco‐Friendly Nano‐Factory for Sustainable Inorganic Waste Management: Current Advancements, Challenges, and Opportunities

**DOI:** 10.1002/open.202500104

**Published:** 2025-05-15

**Authors:** Adhish Jaiswal, S. Irudhaya Raj, Adegoke Isiaka Adetunji, Latifa Negadi, Sangeeta Singh, Kaniki Tumba, Indra Bahadur, Imran Uddin

**Affiliations:** ^1^ Department of Chemistry University of Lucknow Lucknow Uttar Pradesh 226007 India; ^2^ Department of Chemistry Indira Gandhi National Tribal University Amarkantak Madhya Pradesh 484887 India; ^3^ Institute for Water and Wastewater Technology Durban University of Technology Durban 4000 South Africa; ^4^ LATA2M Laboratoire de Thermodynamique Appliquée et Modélisation Moléculaire University of Tlemcen Post Office Box 119 Tlemcen 13000 Algeria; ^5^ Thermodynamics‐Materials‐Separations Research Group Department of Chemical Engineering Mangosuthu University of Technology Umlazi Durban 4031 South Africa; ^6^ Department of Chemistry North‐West University (Mafikeng Campus) Private Bag X2046 Mmabatho 2735 South Africa; ^7^ Department of Conservative Dentistry and Endodontics Saveetha Dental College and Hospital Saveetha Institute of Medical and Technical Sciences (SIMATS) Saveetha University Chennai, Tamil Nadu 600077 India

**Keywords:** bioleachings, biosynthesiss, e‐wastes, inorganic wastes, microorganisms, nanoparticles

## Abstract

Inorganic waste management and metal recovery technology pose significant challenges to the global research community and policymakers. Bioleaching is an economical, eco‐friendly, and sustainable technology used for extracting metals and nanoparticles from inorganic waste streams, including e‐waste, fly ash, ore, spent batteries, and petroleum catalysts. Valuable metals are recovered from these waste materials by microbial action. Bioleaching occurs by redoxolysis, acidolysis, and complexolysis processes. The present review provides insights into global trends and hazards of e‐waste while also discussing the bioleaching of metals and nanoparticles from various inorganic wastes. In addition, the review focuses on the mechanistic pathway of the bioleaching process and computational aspects, such as the response surface method employed for enhanced recovery of metals from solid waste materials. Various physicochemical and biological parameters influencing metal bioleaching, as well as economic impacts and challenges affecting the bioleaching of metals from solid wastes, are discussed.

## Graphical abstract

The review explores the latest updates and information about the global trends and hazards of e‐waste and also discusses the bioleaching of metals and nanoparticles from various inorganic wastes. It also focuses on the mechanistic pathway of the bioleaching process and computational aspects.

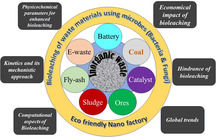

## 1 Introduction

**Figure 1:**
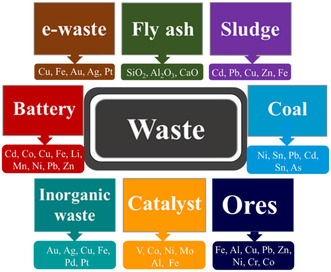
Schematic diagrams depicting different waste materials containing valuable metals.

The global rise in population density, coupled with rapid urbanization and industrialization, has tremendously increased the rate of waste generation.^[^ ^1^, ^2^ ^]^ Uncontrolled dumping of waste materials from various industries pollutes groundwater and soil with harmful substances emitted from the indiscriminate disposal of solid waste.^[^ ^3^, ^4^ ^]^ This further deteriorates soil fertility, leading to nutrient deficiencies in plants.^[^ ^5^ ^]^ Due to low population density and modest use of natural resources, mankind generated very little waste in previous eras.^[^ ^6^ ^]^ The dawn of the Industrial Revolution emerged as a significant challenge in waste management and has become a critical issue to be addressed. Furthermore, the rise in living standards and economic development has increased the quantity and complexity of these waste materials. People and the environment are in danger because of much waste from industries and biomedical waste released into the environment as many sectors grow and healthcare needs rise. Metal‐containing waste includes electronic waste (e‐waste), fly ash, sludge, coal mines, power plants, catalysts, batteries, etc. (**Figure** **1**).

However, the rapid growth of electronics has resulted in the creation of e‐waste, which is made up of abandoned electronic items.^[^ ^7^ ^]^ As a result, the primary goal of waste management is to guarantee safe clearance that results in the recovery of valuable metals. Recycling of solid waste materials through mechanical, hydrometallurgical, and pyrometallurgical methods has been reported.^[^ ^8^, ^9^ ^]^ In addition, methods such as ultrasonic extraction and electrodialysis are also employed for metal extraction. These physical and chemical recycling techniques for solid waste management produce hazardous gases that pollute the environment.^[^ ^10^, ^11^, ^12^ ^]^ Hydrometallurgical operations might pose an environmental danger since harmful chemicals are used and many by‐products are produced. These are costly procedures due to the need for additional investment in a more efficient setup and high energy usage.^[^ ^13^ ^]^ Therefore, an alternative approach to alleviate the aforementioned challenges is the use of microbes for the extraction of metals from inorganic waste.

**Figure 2:**
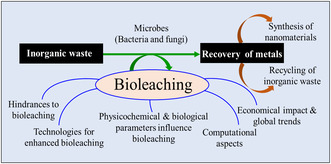
Schematic diagrams illustrating different aspects of bioleaching.

Bioleaching is an innovative and promising technology for treating inorganic waste in comparison to existing methods such as incineration, landfill, and gasification, which pose environmental and health hazards and generate a considerable number of by‐products^[^ ^14^, ^15^, ^16^ ^]^ (**Figure** **2**).

In this technique, waste materials are treated using microorganisms (bacteria, fungi, and yeasts).^[^ ^17^, ^18^, ^19^ ^]^ These microorganisms can produce successful bioleaching of metals like silicon, copper, silver, gold, aluminum, lead, and tin from inorganic solid wastes. The bioleaching of metals from these waste materials and obtaining the metals in the pure nano form could be more beneficial.^[^ ^20^, ^21^ ^]^ This is an economically feasible method that consumes less energy. The efficiency of bioleaching is dependent on factors such as nutrients, availability of O_2_ and CO_2_, pH, temperature, mineral substrate, surfactants, and organic extracts.^[^ ^22^ ^]^ More so, microbiological factors, such as microbial diversity, population density, and microbial activity, must be considered for enhanced recovery of metals from different waste materials and minimization of jarosite formation.^[^ ^23^ ^]^ In general, the availability of favorable conditions significantly influences the ease with which the bioleaching process may be facilitated.^[^ ^24^, ^25^ ^]^ The conversion of organic or inorganic acids into protons, oxidation and reduction processes, and the release of complexing agents are all aspects involved in the mobilization and leaching efficiency of microbes from solid substrates.^[^ ^26^, ^27^, ^28^ ^]^ This technique can be carried out in shake flasks or bioreactors, depending on the particle size of metal‐laden solid waste materials.^[^ ^29^ ^]^ Large‐scale operations are carried out ex situ with the aid of vat, dump and heap, and agitated tank reactor methods.^[^ ^30^ ^]^ Thus, this review focuses on strategies employed by microorganisms for the recovery of metals and the co‐synthesis of nanoparticles from inorganic waste materials.

## 2 E‐Waste: Global Trends and Hazards

**Figure 3:**
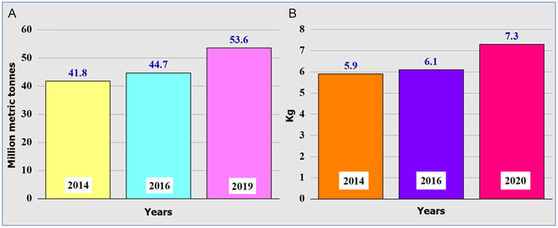
Schematic diagrams showing global A) annual and B) per capital e‐waste generation.

Electronic waste (e‐waste) is defined as any piece of electrical and electronic equipment that has reached the end of its useful life.^[^ ^31^ ^]^ This waste material is on a significant rise in both developing and developed countries. Unfortunately, the most common method for the disposal of solid waste is landfilling.^[^ ^32^ ^]^ It is considered detrimental to human health and pollutes the environment, although there is a lot of potential for recycling and recovering metals from it. However, globally, e‐waste is mismanaged, as evidenced by a low rate of recycling and recovery and a high rate of illegal trade from developed to developing countries.^[^ ^10^ ^]^ The primary conclusions of the United Nations University's (UNU) Global E‐waste Monitor Report 2020 include the following. 1) In 2014, 2016, and 2019, global e‐waste generation reached 41.8, 44.7, and 53.6 million metric tonnes, respectively. It is predicted that by 2030, it will have increased to 82 million metric tonnes (**Figure** **3**A).^[^ ^33^ ^]^ 2) Only 17.4% of global e‐waste was formally collected, treated, and regenerated. 3) Global e‐waste generated per capita was 5.9, 6.1, and 7.3 kg per person for 2014, 2016, and 2020, respectively (Figure 3B). 4) In e‐waste generation, Europe, Oceania, and America ranked first, second, and third, respectively, worldwide with 16.2, 16.1, and 13.3 kg per capital, respectively, whereas Asia and Africa recorded lowest e‐waste generation per capital of 5.6 and 2.5 kg, respectively. 5) The number of countries that have e‐waste policies, laws, or regulations has increased from 61 to 78. 6) Mercury, a toxic additive in e‐waste, is estimated to be 50 tonnes annually. 7) E‐waste consisted majorly of 10.8 metric tonnes of temperature exchange equipment, 13.1 metric tonnes of large equipment, 17.4 metric tonnes of small equipment, as well as 6.7 metric tonnes of screens and monitors, 4.7 metric tonnes of lamps, and 0.9 metric tonnes of small information technology and telecommunication equipment.

**Figure 4:**
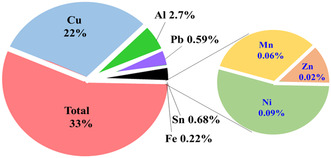
Schematic diagrams elucidating the weight composition of various metals in e‐waste.^[^ ^34^ ^]^

The UNU report estimates that metals such as gold, silver, copper, and platinum are valued at 57 billion dollars, found to be more than the gross domestic product (GDP) of most countries. Hence, these treasured metals are dumped or incinerated rather than collected, recycled, and reused. The recovery of various metals from e‐waste is illustrated in **Figure** **4**.^[^ ^34^ ^]^

In 2014 and 2016, the global and selected countries in terms of e‐waste production are shown in **Table** **1**.^[^ ^35^, ^36^ ^]^

**Table 1:** Global e‐waste production.

Country/region	Per capital e‐waste production	Total e‐waste production [million tonnes]
World (2014 data)	5.9	41.8
World (2016 data)	6.1	44.7
World (2019 data)	–	53.6
World (2020 data)	7.3	–
World (2030 projected)	–	74.0
United Kingdom	23.5	1.50
USA	22.1	7
Germany	21.7	1.77
Hong Kong	21.5	0.16
Canada	20.4	0.72
Australia	20.1	0.46
Singapore	19.6	0.11
European Union	18.7	9.50
Taiwan	18.6	0.44
Japan	17.3	2.20
South Korea	15.9	0.80
Malaysia	7.6	0.23
Brazil	7.0	1.40
Argentina	7.0	0.29
South Africa	6.6	0.35
China	4.4	6.00
Sri Lanka	4.2	0.09
India	1.3	1.60
Nigeria	1.3	0.22
Zambia	0.9	0.01

Developed countries such as the USA and EU generate high e‐waste production when compared to developing countries. However, the per capita e‐waste production by developing countries is comparable to that of developed countries. The United Nations Association (UNA) report in 2017 estimated 7 million, 3 million, and 1.3 million tonnes of e‐waste generated in North America, South America, and Central America, respectively.^[^ ^36^ ^]^ It is reported that e‐waste generated in Pakistan is 38 kt per year; however, over 50 kt of e‐waste is imported as scrap.^[^ ^37^ ^]^ GPS tracking study revealed that 6 of 17 monitors were illegally smuggled from California to China, and 69 of 205 waste electric and electronic equipment items were from the US to 11 different countries.^[^ ^38^ ^]^

According to the UN's fourth Global E‐waste Monitor report in 2024, the worldwide generation of e‐waste is increasing five times faster than documented e‐waste recycling. In addition, about 62 million tonnes of e‐waste were produced in 2022. The annual e‐waste generation is rising by 2.6 million tonnes.^[^ ^39^ ^]^ The e‐waste contains several hazardous metals, which are toxic and carcinogenic to living organisms and ecosystems (**Table** **2**).^[^ ^38^ ^]^

**Table 2:** Sources and effects of metals in e‐waste.

Metal	Source	Effect	Reference
Hg	Lighting devices of cold cathode fluorescent lamps, monitors, flat screen displays, cathode ray tubes, and PCBs	Autoimmune diseases, hypertension, and acute damage to kidneys, lungs, and eyes	[4, 196]
Pb	Batteries, cathode ray tubes (CRTs), monitors, PCBs, bulbs, and lamps	Alzheimer's disease, neurodegenerative diseases, kidney damage, and bone growth	[197]
Cd	Rechargeable batteries, mobile phones, toner photocopy machines, CRT, PCBs, cathode ray tubes, switches, and electronic chips	Renal toxicity, weight loss, lung cancer, osteoporosis, and hyperuricemia	[134]
Cr	Data tapes, floppy disks, and anticorrosion coatings	Reproductive and embryo toxicity, lung cancer, dermatitis, and skin ulcers	[198]
Ni	Ni–Cd batteries, cathode ray tubes	Lung cancer, cardiovascular disease, and oxidative stress	[136]
Li	Li batteries	Inhibition of inositol monophosphatase, nausea, diarrhea, dizziness and fatigue	[199]
Zn	Metal coatings, cathode ray tubes, and batteries	Gastrointestinal, respiratory, and neuronal disorders and prostate cancer	[200]
Ba	Cathode ray tubes and fluorescent lamps	Cardiovascular and kidney diseases, gastrointestinal dysfunction, paralysis, and mental disorders	[201]
Be	Power supply boxes, computers, and electronics	It damages the liver, heart, kidneys, and nervous system	[202]

Hence, it is vital to recover these metals before disposal. The electrical industry has shown specific enhancement in using low‐toxic chemicals and metals; however, e‐waste disposal and handling are of great concern. Sustainable waste management by the 3 R's (reduce, reuse, and recycle), policies of government and environmental agencies, need considerable attention in the increased e‐waste production. Thus, efficient e‐waste management remains a challenge that needs attention in developed and developing countries.

## 3 Waste Material Processing: A General Scenario

**Figure 5:**
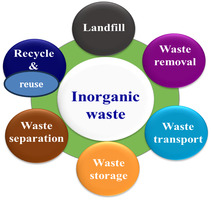
Schematic diagrams showing strategies for management of inorganic waste.

In general, the treatment of waste materials (organic or inorganic) is based on the composition of waste products.^[^ ^40^ ^]^ For instance, organic waste (e.g., kitchen waste) are more easily handled since most are derived from households. These wastes are biodegradable; hence, techniques like incineration, landfilling, and composting are commonly practiced worldwide for treatment. Inorganic waste consists of materials whose biodegradation is difficult and takes a very long time for degradation.^[^ ^41^ ^]^ Most of the inorganic waste comes from nonrenewable natural resources like petroleum products, metal mining industries, and electronic industries.^[^ ^42^ ^]^ These wastes must be dealt with carefully since most are hazardous. Most of these products are treated by recovery methods, which involve extracting valuable materials from waste products, recycling techniques, and converting the waste materials into new products. The general methods of inorganic waste management include landfilling, waste removal, waste transportation, storage, waste separation, waste treatment, recycling, and reuse (**Figure** **5**).

## 4 Bioleaching of Metals from Solid Waste Materials

Metals are important today and are linked with industrial development and improved living standards.^[^ ^43^ ^]^ Electronic waste has been generated worldwide, and disposal concerns have increased dramatically with continuous growth. According to the United Nations Environment Programme (UNEP), about 10% of the global 50 million tonnes of electronic waste is recycled yearly.^[^ ^44^ ^]^ Heavy metals present in the waste materials are discharged into the environment by various industries, including mining, electronics, metallurgical, metal finishing, electroplating, etc. These heavy metals are found in garbage from the mining and metal industries as well as in high concentrations in the waste from power plants, waste incineration facilities, and municipal solid waste (MSW). The extraction of such metals using a simple and cost‐effective method continues to be a challenge.

Bioleaching is referred to as a green, sustainable, and promising technology for the recovery of metals from low‐grade solid wastes.^[^ ^45^ ^]^ This approach is economical based on energy and material requirements and generates fewer waste byproducts.^[^ ^46^ ^]^ In addition, bioleaching permits easy and selective recovery of metals from solid matrices.^[^ ^9^ ^]^ The pretreatment of waste follows two beneficial effects, namely the removal of toxic substances from the waste and the recovery of valuable metals from waste materials. The major industrial wastes are fly ash, electronic scraps, spent refinery catalysts, sewage sludge, lithium‐ion batteries (LIBs), etc. The heavy metals present in these solid wastes mostly include Pb, V, Ni, Mo, Cu, Co, Cr, and Zn.^[^ ^47^ ^]^ Microbes, such as bacteria and fungi, present in the natural environment are generally involved in bioleaching. These organisms are generally categorized as chemolithotrophic bacteria, heterotrophic bacteria, and fungi. The chemolithotrophic bacteria oxidize Fe^2+^ to Fe^3+^, or S^0^ to H_2_SO_4_ during metal bioleaching. In other words, these bacteria enable the oxidation of insoluble inorganic metals into soluble forms.^[^ ^48^ ^]^ The produced Fe^3+^ and H_2_SO_4_ act as lixiviants for the extraction of metals from the solid matrix through acidolysis and redoxolysis.^[^ ^49^, ^50^ ^]^ This category of organisms can thrive well at acidic pH and high metal concentrations. In contrast, heterotrophic bacteria and fungi secrete lixiviants (e.g., HCN and acids), which facilitate the mobilization of metals from solid wastes.^[^ ^51^, ^52^ ^]^ Remarkably, cyanogenic bacteria produce HCN under alkaline conditions in the presence of glycine for the recovery of precious metals from e‐waste.^[^ ^53^ ^]^ The HCN facilitates the dissolution of metals from the solid matrix by forming a soluble metal–cyanide complex, which triggers the recovery of precious metals.^[^ ^54^, ^55^ ^]^ Metal recovery from solid waste materials by microorganisms involves three different techniques, namely, one‐step bioleaching, two‐step bioleaching, and spent medium bioleaching.^[^ ^56^ ^]^

Based on their temperature requirements, these microbes exist as mesophiles or thermophiles. These microorganisms are significant in mobilizing and leaching different metals from solid materials and persist in various applications in the presence of toxic metals in solid waste. Metals are mobilized from solid waste in diverse ways using single or mixed microbial cultures.^[^ ^54^ ^]^ For instance, *Pseudomonas aeruginosa* was employed as a single culture in metal mobilization, whereas a blend of *Chromobacterium violaceum* and *Pseudomonas aeruginosa* showed the highest metal mobilization.^[^ ^57^ ^]^ In addition, during the leaching of heavy metals using bacteria from anaerobically digested sludge, mixed cultures of *Thiobacillus thiooxidans* and *Thiobacillus ferrooxidans* outperformed single cultures in solubilizing rates.^[^ ^27^ ^]^ Bacterial activity affects the chemical bonds of pollutants in waste, leading to increased solubility of the pollutant.^[^ ^58^ ^]^ The metals are effectively leached out and converted to nanosized particles encapsulated with protein via fungal‐mediated bioleaching. The amino acids of proteins and enzymes played a crucial role in fly ash bioleaching by responding to stress conditions.^[^ ^59^ ^]^ The fungus (*Fusarium oxysporum*) was effective in bioleaching silica nanoparticles derived from zircon and white sand.^[^ ^60^, ^61^ ^]^ *Humicola* sp. has been used in bioleaching to obtain silicate nanoparticles from the glass surface.^[^ ^62^ ^]^ Nutrient, inoculum volume, and medium composition, which enable optimal microbial growth and allow the production of essential metabolites that help in bioleaching, are the best conditions for maximum metal extraction. In addition, adequate oxygen and carbon dioxide supply coupled with optimum pH and temperature enhances microbial growth and bioleaching efficiency.^[^ ^63^ ^]^ Furthermore, the metal complex's chemical state, substrate composition, and particle size influence the effectiveness and rate of the bioleaching process. Microorganisms mobilize and leach metals from solid waste materials using three principles, namely, redoxolysis (oxidation and reduction processes), acidolysis (the generation of organic or inorganic acids), and complexolysis (the excretion of complexing agents).^[^ ^64^ ^]^ In the case of complexolysis, the desired metals from a solid matrix (e.g., ore) form a complex with appropriate ligands (e.g., cyanide), leading to the recovery of metals from the inorganic source.^[^ ^56^, ^65^, ^66^ ^]^ For instance, the secretion of Fe‐chelating compounds by certain microorganisms facilitates the recovery of ferric ions. In addition, organic acids from some fungi produce protons, which enhance the complexing aptitude during bioleaching.^[^ ^51^ ^]^ Choi et al.^[^ ^67^ ^]^ reported that bioleaching of copper from printed circuit boards (PCBs) by *Acidithiobacillus ferrooxidans* followed the metal sulfide leaching process. Sulfuric acid is also the principal inorganic acid produced by sulfur‐breaking bacteria such as the *Acidithiobacillus* species.^[^ ^68^, ^69^, ^70^ ^]^ Organic acidolysis, complex, and chelate formation are all affected by the organic acids generated by bacterial and fungal metabolism. The redoxolysis generates organic and inorganic acids from solid waste materials and secretes complex agents.^[^ ^71^ ^]^

## 5 Coordination Chemistry of Bioleaching of Metals from Solid Waste Materials

The release of different metabolites by microbes, including organic acids, siderophores, and other complexing agents that can coordinate with metal ions, is a significant factor in coordination chemistry in bioleaching.^[^ ^72^ ^]^ Oxalic acid, citric acid, and gluconic acid are among the organic acids frequently produced by microorganisms involved in bioleaching.^[^ ^73^, ^74^ ^]^ With the help of functional groups like carboxylates, these organic acids can interact with metal ions to form metal complexes and increase the solubility of metal.^[^ ^75^, ^76^ ^]^ Microorganisms produce tiny molecules called siderophores with a high affinity for chelating iron.^[^ ^77^, ^78^, ^79^ ^]^ Along with their coordination ability, siderophores can also aid in solubilizing and extracting other metal ions like copper, zinc, and nickel.^[^ ^79^ ^]^ Functional groups (such as carboxyl and amino) found in microbial biofilms and extracellular polymeric substances (EPS) bind to metal ions.^[^ ^80^ ^]^ By coordinating with metal ions, EPS in biofilms can affect metal speciation and mobility. Redox processes in which microorganisms take part can change the oxidation states of metals. An essential component of the coordination chemistry of bioleaching is the transfer of electrons between microorganisms and metal ions.^[^ ^81^ ^]^ Therefore, improving metal recovery from ores, designing effective bioleaching strategies, and optimizing process conditions all depend on an understanding of the coordination chemistry of bioleaching. Researchers employ a range of analytical and spectroscopic techniques to understand the intricate relationships that arise during bioleaching between microbial metabolites and metal ions.^[^ ^77^, ^78^, ^79^, ^80^, ^81^ ^]^

Coordination chemistry plays a crucial role in the metallurgy/extraction of various metals, including precious metals, heavy metals, and rare‐earth elements present in a variety of inorganic solid matrices, such as electronic waste, LIBs, fly ash, low‐grade ore, and waste rock dump.^[^ ^82^ ^]^ These metals exist in complex chemical compounds associated with many surrounding bound molecules or ions (ligands) via coordinate covalent bonds.^[^ ^83^ ^]^ The recovery of metals from complexes by a biological technique using microorganisms is called bioleaching. The various applications of coordination chemistry in metal extraction are highlighted as follows.^[^ ^84^, ^85^, ^86^ ^]^

1) Bioleaching of precious metals (such as gold, silver, and platinum), in which HCN produced as a secondary metabolite by microorganisms dissolves the precious metals from mineral concentrates or e‐waste through the formation of a soluble metal–cyanide complex, which facilitates extraction of the metals. The dissolution of precious metals by cyanide consists of anodic (1) and cathodic (2) reactions, which are summarized in Equation (3) illustrated as follows
(1)

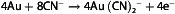

(2) $$O_{2}+{2H}_{2}{O+4e}^{-}\to {4\text{OH}}^{‐}$$
(3)

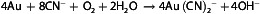

  In Equation (3), the complexation of Au^+^ ion by a ligand (CN^−^) forms a coordination complex, Au (CN)_2_^−^.
2) Cyanide complex–containing solution is treated with zinc when gold is precipitated, as shown in Equation (4)
(4) $$2{\left[\text{Au }{\left(\text{CN}\right)}_{2}\right]}^{-}+\text{Zn}\to {\left[\text{Zn }{\left(\text{CN}\right)}_{4}\right]}^{2-}+2\text{Au}$$
3) During chemical leaching (hydrometallurgy) of sulfide ores of Co, Ni, Cu, Zn, and Cd using ammonia, the metal components dissolve as ammine complexes, leaving the associated Fe as a residue. This is illustrated in Equation (5)
(5)

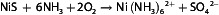

  The Ni is isolated from the leach liquor by precipitation using H_2_.
4) KCN is used to extract silver from Ag_2_S in the form of a cyanide complex and then reduce it with zinc as shown in Equation (6) and (7)
(6) $${4\text{KCN}+\text{Ag}}_{2}S\to \text{2K}\left[\text{Ag }{\left(\text{CN}\right)}_{2}\right]+K_{2}S$$
(7) $$2K\left[\text{Ag }{\left(\text{CN}\right)}_{2}\right]+\text{Zn}\to K_{2}\left[\text{Zn }{\left(\text{CN}\right)}_{4}\right]+2\text{Ag}$$
5) Purification of some metals can be achieved through complex formation. For instance, in the Mond process, impure nickel is converted into [Ni (CO)_4_], which is later decomposed to yield pure nickel. The formation of soluble complexes of these metals enables their separation from those that form insoluble species and the subsequent isolation of the constituent metals by suitable precipitation methods.

## 6 Mechanistic Approach for Bioleaching of Metals

**Figure 6:**
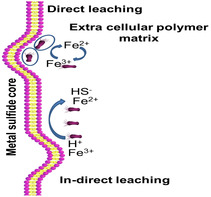
Schematic diagrams showing direct versus in‐direct bioleaching.

Bioleaching is accomplished in various ways, the most common of which are physical procedures (also known as direct bioleaching) and chemical methods (also known as indirect bioleaching).^[^ ^26^ ^]^ In direct bioleaching, the microorganisms react directly on the metal surface for the oxidation of sulfide to sulfate through many enzymatic steps (**Figure** **6**). The bacteria generate an extracellular polymeric layer between the cell membrane and the sulfide.^[^ ^87^ ^]^ In addition, the microbes attach to a surface region that has a weakness in the crystal structure. Metal dissolution from the solid surface takes place via electrochemical reactions.^[^ ^22^ ^]^

In indirect bioleaching, the microbes secrete certain leaching agents (lixiviant) (e.g., Fe^3+^), which facilitate the dissolution of metals from the solid matrix without requiring microbial attachment.^[^ ^88^ ^]^ The lixiviants chemically oxidize the sulfide minerals in an acidic environment (pH 5.0) for the release of ferrous iron. The ferrous iron can be reoxidized to ferric iron by iron‐oxidizing organisms (such as *Thiobacillus ferrooxidans*) used in the bioleaching process. The whole procedure does not need cell adhesion to the solid substrate surface.^[^ ^68^ ^]^

The two mechanisms are summarized in the following equations

1) Direct bioleaching
(8)

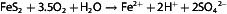

(9) $${2\text{Fe}}^{2+}+0.5O_{2}{+2H}^{+}\to {\text{2Fe}}^{\text{3+}}+H_{2}O$$
2) Indirect bioleaching
(10)

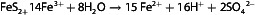

(11) $${\text{MS}+\text{2Fe}}^{3+}\to M^{2+}+S^{0}+{\text{2Fe}}^{2+}$$
(12)

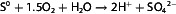

## 7 Applications of Bioleaching

Bioleaching is recognized as an economical, eco‐friendly, and sustainable technology for the recovery of metals since it requires no capital investment, labor, or energy.^[^ ^89^ ^]^ The microbial agents isolated from extreme and toxic environments have inbuilt biosynthetic mechanisms to degrade any compound. In recent years, various researchers have demonstrated the ability to manipulate microorganisms and their application in bioleaching and remediation processes.^[^ ^90^, ^91^ ^]^

### 7.1 Bioleaching of Metals from Electronic Scraps

Technological developments and modern business marketing strategies continuously opened new platforms for innovative research products that quickly swapped outdated electronic versions. Thus, every year, high percentages of electronic waste are accumulated globally. According to the United Nations Environment Programme, e‐waste (20–50 million) is produced every year, and the amount of e‐waste grows three times more than other forms of municipal garbage.^[^ ^92^ ^]^ To overcome this challenge, several studies on the bioleaching of metals from electronic trash using microorganisms have been published in recent years.^[^ ^93^ ^]^ Sulfur‐ and iron‐oxidizing bacteria (including *Acidithiobacillus ferrooxidans*, *Acidithiobacillus thiooxidans*, *Thermoplasma acidophilum*, *Leptospirillum ferriphilum*, *Sulfobacillus thermosulfidooxidans*, etc.) cyanogenic bacteria (such as *Bacillus megaterium*, *Pseudomonas* sp., and *Chromobacterium violaceum*), and heterotrophic fungi (belonging to the genera *Trichoderma*, *Candida*, *Saccharomyces*, *Aspergillus*, and *Penicillium*) are well known for bioleaching of metals from e‐waste.^[^ ^9^, ^25^, ^28^, ^51^, ^94^, ^95^ ^]^ Remarkably, Benzal et al.^[^ ^96^ ^]^ employed *Acidithiobacillus ferrooxidans* for the recovery of metal from PCB of mobile phone at 30 °C (130 rpm) using a two‐step bioleaching technique. Maximum copper removal efficiency of 95%–100% was recorded. In addition, optimum 98% Cu and 82% Ni were reported by Kadivar et al.^[^ ^97^ ^]^ during bioleaching of metals from discarded mobile phone PCBs by *Acidithiobacillus thiooxidans* at 30 °C and 160 rpm for 3 d. Kumar et al.^[^ ^98^ ^]^ employed *Pseudomonas balearica* SAE1 for the bioleaching of metals from waste PCBs at optimum conditions of pulp density 10 g L^−1^, glycine concentration 5 g L^−1^, pH 9.0, and 30 °C. Maximum removal efficiencies of 68.5% Au and 33.8% Ag were reported. Similarly, Sahni et al.^[^ ^99^ ^]^ recovered 13.79% Cu, 0.44% Au, and 2.55% Ag from SIM card waste using *Chromobacterium violaceum* MTCC 2656 at pH 9.0, pulp density 10 g L^−1^, glycine concentration 5 g L^−1^, 30 °C, and 150 rpm for 7 d. In addition, Faraji et al.^[^ ^100^ ^]^ recovered 64% Cu, 44% Ni, and 99% Zn from waste PCB by *Aspergillus niger* at particle size <300 μm, pulp density 0.5–20 g L^−1^, 25 °C, 120 rpm, and pH 5.0. Arshadi et al.^[^ ^101^ ^]^ employed *Penicillium simplicissimum* for the extraction of metals from spent PCB. Maximum removal efficiencies of 90% Cu and 89% Ni were recorded at 30 °C, 130 rpm, and a pulp density of 10 g L^−1^.

**Figure 7:**
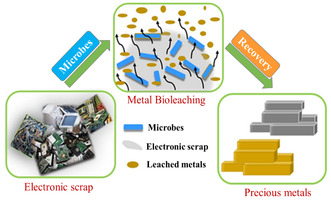
Schematic diagrams depicting recovery of precious metals from electronic waste.

The utilization of microbial consortia to extract metals from e‐waste has been established by many researchers^[^ ^21^, ^91^, ^102^ ^]^ (**Figure** **7**). Karwowska et al.^[^ ^103^ ^]^ used a mixed culture of biosurfactant‐producing bacteria and sulfur‐oxidizing bacteria to remove zinc, cadmium, chromium, nickel, and copper from PCBs. The study revealed the effective removal of zinc and cadmium from the media. However, nickel and copper were removed by 50% compared to the other metals in the medium.

This may be due to many physiological factors such as pH, temperature, bioleaching cultures, and type of metal, which need to be optimized for better results.^[^ ^104^ ^]^ A bacterial consortium obtained from natural acid mine drainage was used by Xiang et al.^[^ ^105^ ^]^ to assess the mobilization of copper from waste PCBs. Within 5 d, copper was primarily extracted via oxidation of ferric ions created by ferrous ion–oxidizing bacteria. Similarly, cocktail of *Acidithiobacillus ferrooxidans* DSMZ583, *Acidithiobacillus thiooxidans* DSMZ9463, and *Leptospirillum ferrooxidans* DSMZ2705 gave maximum removal efficiencies of 97.3% Cu, 55.8% Al, 79.3% Ni, and 66.8% Zn during an experiment involving bioleaching of metals from waste PCB of mobile phone at 30 °C, pH 1.8, 150 rpm, pulp density 10%, inoculum volume 10% (v/v), incubation period of 8 d, and oxidation–reduction potential of >650 mV.^[^ ^106^ ^]^ In addition, a mixed culture of *Leptospirillum ferriphilum* and *Sulfobacillus thermosulfidooxidans* was used by Wu et al.^[^ ^107^ ^]^ for the mobilization of Cu from waste PCBs at 100 g L^−1^ PCB for 9 d. The authors reported ≈94% Cu removal efficiency.

### 7.2 Bioleaching of Metals from Spent LIBs

LIBs are extensively employed as rechargeable energy resources in various applications, including electric vehicles, mobile phones, and other portable electronic devices, due to their rapid charging potential, higher output voltage, longevity, and high energy density.^[^ ^108^, ^109^ ^]^ The rise in the market demand for LIBs reached USD 36.7 billion in 2019, and this is predicted to surge by ≈fourfold to USD 129.3 billion in 2027.^[^ ^110^ ^]^ However, the innovation of new technologies leads to the obsolescence of electronic products. This generates astonishing quantities of LIBs, discarded as waste materials. These batteries are typically classified as hazardous rubbish due to the existence of heavy metals and other potentially harmful chemicals in electronic waste, which frequently results in the recovery of precious gemstones from the waste.^[^ ^111^, ^112^, ^113^ ^]^ The spent LIBs are considered a secondary source of metals, with components higher than those in the concentrated ore.^[^ ^114^ ^]^

The LIB consists of 15%–17% anode, 35% cathode, 25%–30% battery casings, 11%–12% electrolyte, and 5%–6% plastic materials.^[^ ^115^ ^]^ However, lithium cobalt oxide is one of the most desired cathodes employed in a variety of portable electronic devices. The two main metals in discarded LIBs include Co and Li, constituting 30.4% and 10.3% of the overall weight of the spent LIB.^[^ ^115^ ^]^ In addition, other elements, such as Fe, Cu, Al, Mn, and Ni are found in varying quantities in discarded LIBs. Such differences in the metal composition of LIBs are attributed to variations in battery chemistry.^[^ ^66^ ^]^ However, Co is a costlier material than the other elements of the battery. These elements are recovered from the battery using the bioleaching technique.^[^ ^109^ ^]^ Pure culture or consortium of acidophilic bacteria is employed for the removal of precious metals from spent LIBs. For instance, Mishra et al.^[^ ^116^ ^]^ exploited *Acidithiobacillus ferrooxidans* to dissolve metals from waste LIBs. This bacterium utilizes elemental sulfur and ferrous ions as the energy source to produce sulfuric acid and ferric ion metabolites in the leaching medium for extraction. Li et al.^[^ ^117^ ^]^ reported 47.6% Co recovery from spent LIB in the presence of *Acidithiobacillus ferrooxidans*. Naseri et al.^[^ ^118^ ^]^ investigated bioleaching of metals from discarded LIBs using *Acidithiobacillus thiooxidans* PTCC 1717. The authors recorded higher recovery efficiencies of Co 60%, Mn 20%, and Li 99%. In another study, a mixed culture of *Acidithiobacillus ferrooxidans* and *Acidithiobacillus thiooxidans* was utilized for the bioleaching of metals from waste LIBs, resulting in 67% Co and 80% Li.^[^ ^119^ ^]^ A cocktail of thermophilic bacteria, including *Ferroplasma* sp., *Acidithiobacillus caldus*, *Leptosprillum ferriphilum*, and *Sulfobacillus* sp. resulted in the dissolution of 99.7% Ni, 99.9% Co, and 84% Li from discarded laptop LIB.^[^ ^115^ ^]^

Furthermore, a plethora of fungi have been recorded for the bioleaching of metals from spent LIBs. However, *Aspergillus niger* is mostly preferred owing to higher productivity, simple cultivation process, tolerance to many toxic metals, rapid leaching rate, and ability to grow in both acidic and alkaline medium.^[^ ^120^, ^121^ ^]^ Biswal et al.^[^ ^122^ ^]^ carried out an experiment on bioleaching of metals from spent LIBs using *Aspergillus niger* MM1 and *Aspergillus niger* SG1. Maximum removal efficiencies of 80%–82% Co and 100% Li were recorded. In another study, Alavi et al.^[^ ^123^ ^]^ reported 60% Co, 95% Li, as well as ≈80%–98% Ni, Al, and Mn during biorecovery of metals from spent cellphone LIBs. Bahaloo‐Horeh and Mousavi^[^ ^73^ ^]^ reported 64% Co, 100% Li, 100% Cu, 54% Ni, 75% Al, and 77% Mn during bioleaching of metals from discarded LIBs in the presence of *Aspergillus niger* PTCC 5210 at pulp density of 1%–2% (w/v). *Penicillium chrysogenum* PTCC 5037 gave a maximum Li recovery efficiency of 54.6% from spent LIBs at pH 4.5 and pulp density 10% (w/v).^[^ ^124^ ^]^

### 7.3 Bioleaching of Metals from Fly Ash

The disposal of MSW is a source of concern around the globe owing to dwindling landfill space and the development of hazardous leachate from rainfall penetration. To reduce the MSW, all nations commonly follow incineration processes, resulting in the production of a finely divided residue called “fly ash”. Fly ash is a hazardous waste generated in large amounts by MSW incinerators worldwide. The volatile toxic metals in the ashes make them hazardous because they build up and become concentrated. According to chemical analyses, the fly ash includes a substantial quantity (2000–20,000 mg kg^−1^) of harmful heavy metals such as aluminum, lead, and zinc.^[^ ^125^ ^]^ As a result, bioleaching is a better alternative and promising technique for the valorization of fly ash.^[^ ^21^ ^]^ For instance, Ramanathan and Ting^[^ ^126^ ^]^ isolated 18 indigenous bacteria tolerant to the alkaline pH of fly ash with bioleaching potential from Pulau Semakau landfill site. According to the results of the analysis of the microbial genome, isolate TRTYP6, belonging to *Alkalibacterium* sp., had the highest fly ash tolerance of up to 20% (w/v) and could grow at pH values ranging from 8.0 to 12.5. The study strain demonstrated optimum (52%) Cu recovery from MSW incineration fly ash. Similarly, *Acidithiobacillus ferrooxidans* DSMZ 3320 recorded highest removal efficiencies of 85% (K), 47% (Na), 92% (Ca), 85% (Mg), 46% (Al), 67% (Zn), 11% (Pb), and 55% (Mg) during bioleaching of metals from municipal waste incineration fly ash following secretion of 0.0289 M sulfuric acid.^[^ ^127^ ^]^ Yang et al.^[^ ^63^ ^]^ investigated the bioleaching of metals from MSW incineration fly ash (70 g L^−1^) using multi‐metal adapted *Aspergillus niger*. The results obtained showed recovery of 45.9% Pb, 49.4% Zn, 64.8% Mn, and 87.4% Cd, following secretion of 256 mmol L^−1^ organic acids after 12 d. Similarly, Wu and Ting^[^ ^128^ ^]^ used *Aspergillus niger* as a bioleaching agent for the removal of metals from fly ash using a two‐step process. The authors reported a significant metal extraction (60%–70% of Cu and Pb; 80%–100% of Al, Mn, and Zn; and 30% of Fe). Park and Liang^[^ ^129^ ^]^ reported the highest leaching efficiencies of 80.9, 79.5, 67.7, and 64.6% for As, Mo, Yb, and Er, respectively, during bioleaching of trace elements and rare‐earth elements from coal fly ash by *Candida bombicola*. In addition, *Aspergillus niger* CGMCC 3.15663 was subjected to secretion of organic acids for the recovery of 30.91% total rare‐earth elements from coal fly ash.^[^ ^130^ ^]^

The use of a consortium of microorganisms is effective with high metal tolerance for the bioleaching of metals from fly ash. For instance, a mixed culture of *Thiobacillus thiooxidans* TM‐32 and *Acidithiobacillus ferrooxidans* ATCC 23270 was employed by Ishigaki et al.^[^ ^131^ ^]^ for the bioleaching of metals from municipal waste incineration fly ash. At 1% ash culture, maximum removal efficiencies were reported as 67% (Cu), 78% (Zn), and 100% (Cr and Cd). In addition, Li et al.^[^ ^13^ ^]^ investigated the effectiveness of multi‐metal removal from plant incineration fly ash using a cocktail of *Sulfobacillus thermosulfidooxidans*, *Ferroplasma thermophilum*, and *Leptosprillum ferriphilum*. The bioleaching results showed higher recovery of Zn (88.7%), Pb (13.8%), Mn (99.9%), and Cd (93.2%) at an optimum Fe^2+^ concentration 6 g L^−1^, pulp density of 15% (w/v), and pH 1.8.

### 7.4 Co‐synthesis of Nanoparticles from Solid Waste Materials

**Figure 8:**
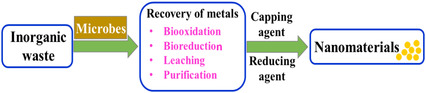
Schematic diagrams illustrating synthesis of nanomaterials from inorganic solid waste.

This new method revolutionized the concept of cost‐effective technology by extracting metals from waste material while also synthesizing nanoparticles of important biomedical applications (**Figure** **8**).

This is an environmentally friendly technique that involves the application of microorganisms (e.g., fungi and bacteria) for the biosynthesis of nanoparticles.^[^ ^132^ ^]^ However, this technique is faced with some challenges, including long duration, less control of nanoparticle size, possible culture contamination, and difficulty in large‐scale production.^[^ ^90^ ^]^ Nanoparticle synthesis is influenced by the capping agents, pH, temperature, pressure, reaction time, distribution size, etc.,^[^ ^90^ ^]^ According to Khan et al.^[^ ^17^ ^]^ for the first time, they have demonstrated the ability of the mesophilic fungus *Fusarium oxysporum* to extracellularly bioleach highly crystalline, fluorescent, stable, water‐soluble, and protein‐capped silica nanoparticles from fly ash at ambient conditions within 24 h. This is attributed to the secretion of enzymes and natural protein capping during fly ash bioleaching. Similarly, the authors have reported the bioleaching of brookite‐type TiO_2_ nanoparticles from anatase‐type bulk TiO_2_. Under visible light, these brookite‐type TiO_2_ nanoparticles have been found to exhibit extremely significant antibacterial activity. In addition, spherical‐shaped aggregated clusters of silica nanoparticles (40–80 nm) were synthesized by Fulekar and Yadav^[^ ^133^ ^]^ following incubation of *Fusarium oxysporium* supernatant and sodium silicate at 28 °C for 2–3 d. In this experiment, leached Si from fly ash by the hydrolytic enzymes of *Fusarium oxysporium*, formed water‐soluble silica nanoparticles. Bansal et al.^[^ ^61^ ^]^ utilized *Fusarium oxysporum* to selectively and extracellularly bioleach silica from zircon sand, resulting in the synthesis of crystalline nanoparticles (2–10 nm) capped by stabilizing proteins.^[^ ^134^ ^]^ When exposed to rice husk, *Fusarium oxysporum* leaches out massive volumes of crystalline silica particles at room temperature.^[^ ^135^ ^]^ Silicate nanoparticles were produced by bioleaching glass using *Humicola* sp. at 50 °C with accompanying surface modification.^[^ ^136^ ^]^ Furthermore, silica nanoparticles extracellularly secreted through bioleaching of sand by *Fusarium oxysporium* demonstrated nematocidal potential against *Meloidogyne javanica* (soil‐borne parasite).^[^ ^137^ ^]^

The bioleaching of value‐added nanoparticles from e‐waste and tailing ores has attracted the attention of many researchers for sustainable development and circular bioeconomy. For instance, for the first time, Maass et al.^[^ ^138^ ^]^ reported biosynthesis of stable and iron oxide nanoparticles from coal tailings by *Rhodococcus erythropolis*. Wong‐Pinto et al.^[^ ^139^ ^]^ and Subbaiyan et al.^[^ ^132^ ^]^ synthesized copper nanoparticles from tailings ore and e‐waste using *Pseudomonas stutzeri* and lichen‐associated bacteria (*Parmotrema recticulatum* and *Parmotrema tintorum*), respectively. In addition, ferrous nanoparticles from e‐waste demonstrated antifouling properties.^[^ ^132^ ^]^ Bioleaching of bauxite ore to produce antibacterial alumina nanoparticles was also investigated.^[^ ^140^ ^]^

## 8 Computational Aspects of Bioleaching

The bioleaching of metals is a labor‐intensive procedure in which several variables influence the kinetics of the process as well as the metabolic activity of microorganisms. In bioleaching, pH and temperature are the most important variables to consider. Other factors include composition of the sample (including the abundance of heavy metals and trace elements), solid‐to‐liquid ratio, the microorganism's resistance to metal ions, and the amounts of biomaterials released by the microorganisms.^[^ ^141^, ^142^, ^143^ ^]^ All these parameters affect the whole process quantitatively and qualitatively in terms of the yields and purity, respectively, of the recovered metals. So, the optimization of the process, computation of the relationship between influencing factors, and simulation of influencing parameters are the critical strategies to enhance the recovery of metals with purity in such a multiple‐factor system. Here, the statistical approach is advantageous over other conventional methods. The statistical approach is rapid and can determine the interactions between the factors that affect the bioleaching process.^[^ ^144^ ^]^ The statistical approach combined with the mathematical model can be utilized to simulate the parameters and the statistical evaluation of the experiment.^[^ ^91^ ^]^

### 8.1 Recovery of Metals by Response Surface Method

Significant computational studies of bioleaching have been carried out by the response surface methodology (RSM) and global optimization method, based on the statistical and mathematical approaches, used to optimize the multivariable processes.^[^ ^145^, ^146^, ^147^ ^]^ This technique has been effectively utilized in heavy metal biosorption, fermentation, and waste treatment, among others.^[^ ^148^ ^]^ To predict the interactions among the process variables and process response, RSM optimizes the responses of the influencing factors throughout the operational zone. An empirical mathematical model, namely central composite design (CCD), is the most implemented RSM (**Table** **3**).^[^ ^149^ ^]^ CCD is modelled with the optimal number of experiments and fits using the least square method.

**Table 3:** Quadratic models for the recovery of metals through the bioleaching process.

Model	Second‐order quadratic equation for prediction	Parameter	Reference
Cu recovery model	*Y* = 81.99 + 1.05A + 0.74B + 1.70D + 2.51AB + 1.67AC − 1.70AD + 1.23CD + 1.10A^2^ − 1.84B^2^ + 1.26C^2^ − 7.26D^2^	Y—predicted recovery, 81.99—offset term, A—initial pH, B—initial Fe^3+^ concentration, C—pulp density, D—particle size	[143]
Ni recovery model	*Y* = 83.06 + 5.71A − 1.36B − 2.91C + 0.91D + 3.39AB − 0.076AC − 1.59AD + 1.05BC + 1.60CD − 2.22A^2^ − 1.19B^2^ + 4.06C^2^ − 8.63D^2^	Y—predicted recovery, 83.06—offset term, A—initial pH, B—initial Fe^3+^ concentration, C—pulp density, D—particle size	[143]
Mo recovery model	*Y* = 65.21 + 0.19A + 0.21B − 6.44C − 1.29D − 3.06AB + 0.64AC − 1.96AD − 1.56BC + 2.28BD − 1.04CD + 1.14A^2^ + 0.054B^2^ − 1.24C^2^ + 1.06D^2^	Y—predicted recovery, 65.21—offset term, A—particle size, B—sucrose concentration, C—pulp density, D—pH	[144]
Re recovery model	*Y* = 36.65 − 0.20A + 0.68B − 4.25C − 1.24D − 1.45AB − 0.63AC − 0.57AD − 0.27BC + 0.12BD − 0.31CD − 0.42A^2^ − 0.51B^2^ + 0.84C^2^ + 0.29D^2^	Y—predicted recovery, 36.65—offset term, A—particle size, B—sucrose concentration, C—pulp density, D—pH	[144]
Al recovery model	*Y* = 11.40 − 0.097A + 0.34B − 1.59C − 0.28D − 0.46AB − 0.64AC − 0.15AD + 0.013BC + 0.12BD − 0.33CD − 0.35A^2^ − 0.31B^2^ − 0.32C^2^ − 0.14D^2^	Y—predicted recovery, 11.40—offset term, A—particle size, B—sucrose concentration, C—pulp density, D—pH	[144]
Recovery from Biogenic Sludge	*Y*_M_ = 73.42 − 5.85A + 9.66B − 4.82C − 13.68D − 5.32A^2^ − 5.44B^2^ + 7.39AB + 2.50AC + 4.21BC + 10.12AD + 2.50 CD + 12.50 BD	Y—predicted metal recovery, M—sum of Cu, Zn, Ni, Al, Cd, Cr, Pb, A—acid demand, B—particle size effect, C—pulp density effect, D—bacterial feed formulation	[151]

The CCD method, found to be very effective for process optimization, results in an optimal number of experiments. The optimal number of experiments is computed by equation 2k *+ n*_α_ *+ n*_0_, where *k* is the independent variable, n_α_ is the axial point, and n_0_ is the center point.^[^ ^150^ ^]^ According to Crolla and Keneddy, RSM can be used to study the effects of several parameters as well as the interactions between the parameters at a time.^[^ ^151^ ^]^

Amiri et al.^[^ ^144^ ^]^ selected eleven variables based on their effects on metal recovery from spent refinery catalyst by *Aspergillus niger*, using Plackett–Burman design. These eleven variables were analyzed through the analysis of variance (ANOVA) method. Among the eleven variables, four variables, namely sucrose, particle size, pulp density, and pH, had an ANOVA probability value (*p*‐value) of less than 0.05 (0.015, 0.022, 0.026, and 0.046, respectively). Therefore, these four variables were considered as the significant variables, which have substantial effects on the response. Thereafter, RSM, using a CCD, was employed to determine the optimum levels of the significant variables at five coded levels (−2, −1, 0, 1, 2). The authors determined the significance of coefficients in the quadratic model by calculating the *p*‐values through a statistical approach. Low sucrose content and high particle size are more favorable for molybdenum, nickel, and aluminum recovery. In another study, Arshadi et al.^[^ ^152^ ^]^ applied the second‐order quadratic response surface model, using CCD for optimization of variables for enhanced recovery of Ni and Cu during the bioleaching process. Findings from the study showed that the initial pH positively influenced Ni recovery, while higher pH values favored Ni recovery. The authors reported that an increase in pH and substrate surface area favored the dissolution of the metals from PCBs. The increase in the surface area provides a better accessible surface to the bacteria for enhanced metal bioleaching. The anticipated size factor coefficient was low for both Ni and Cu models. However, particle size interaction had the most significant influence. Pulp density was found to be the most significant factor for the recovery of Cu. Ilyas et al.^[^ ^151^ ^]^ developed a second‐order quadratic model based on the RSM technique for the optimization of bioprocess parameters for enhanced extraction of heavy metals from electronic trash. The particle size, pulp density, bacterial feed composition, and sludge parameters were improved. Findings from this study demonstrate that a decrease in particle size from 212 to 150 μm enhanced metal extraction; however, a further decrease in particle size coupled with an increase in pulp density above 15% decreased process efficiency. In addition, the bacterial feed formulation has a significant impact on the recovery of the metals. Similarly, Kumar et al.^[^ ^98^ ^]^ carried out an experiment on the optimization of biorecovery of metals from computer PCB using CCD of RSM. Maximum removal efficiencies of gold (73.9%) and silver (41.6%) were recorded at optimum pH, temperature, glycine concentration, and pulp density of 8.6, 31.2 °C, 6.8 g L^−1^, and 5 g L^−1^, respectively. Trivedi and Hait^[^ ^153^ ^]^ recovered highest Cu (100%), Pb (40%), Ni (70%), and Zn (100%) from spent cellphone PCBs by *Aspergillus niger*, using Box–Behnken design of RSM at optimum shaking speed, glucose oxidase concentration, pulp density, and Fe^2+^ concentration of 335 rpm, 300 U L^−1^, 1 g L^−1^, and 10 mM, respectively.

Motaghed et al.^[^ ^102^ ^]^ employed RSM for the optimization of bioprocess parameters for enhanced recovery of metals from spent refinery catalyst using *Bacillus megaterium*. At optimum values of glycine (12.8 g L^−1^) and pulp density (4%), maximum removal efficiencies of 15.7% Pt and 98% Re were recorded. Shahrabi‐Farahani et al.^[^ ^154^ ^]^ investigated the bioleaching of metals from hydrocracking spent catalyst by *Acidithiobacillus thiooxidans* using CCD of RSM. Enhanced recovery of Al (15%), Ni (37%), and Mo (87%) was reported at optimum pulp density, particle size, and aeration of 0.9% (w/v), 60.7 μm, and 209 mL min^−1^, respectively. Similarly, CCD of RSM was employed by Srichandan et al.^[^ ^155^ ^]^ for the recovery of metals from spent refinery catalysts using *Acidithiobacillus thiooxidans*. The authors recorded maximum removal efficiencies of 44% (Al), 94% (V), 34% (Mo), and 93% (Ni) at optimum conditions of pH (1.5), pulp density (1%), and sulfur concentration (1.5%). In addition, Gholami et al.^[^ ^141^ ^]^ recorded highest Co, Mo, and Ni recovery of 71, 69, and 46% at optimum conditions of pulp density 2 g L^−1^, 115 rpm, 31.8 °C, inoculum volume 12%, and pH 5.0 during bioleaching of metals from spent catalysts using *Aspergillus niger*.

## 9 Parameters that Influence Bioleaching of Metals from Inorganic Waste Materials

**Figure 9:**
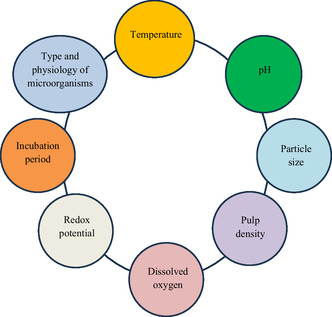
Schematic diagrams showing various physicochemical and microbiological parameters that influence bioleaching of metals from inorganic solid waste.

Several physicochemical and microbiological factors affect the growth of microorganisms for the production of suitable metabolites for the bioleaching of metals from inorganic waste (**Figure** **9**).

Bioleaching is controlled by a variety of physical and chemical parameters, including temperature, pH, substrate composition, particle size, pulp density, inoculum volume, O_2_ and CO_2_ supply, incubation period, etc.^[^ ^28^, ^56^, ^109^ ^]^ These parameters are listed in **Table** **4** and **5** and discussed in detail later.

**Table 4:** Influence of physicochemical parameters on bioleaching of metals from inorganic wastes.

Factor	Factor range	Bioleaching Source	Microorganism	Findings	Reference
pH	≈2.4	Sludge	*A. thiooxidans* TS6 and *A. ferrooxidans* LX5	Removal efficiencies of Cu and Cr reached above 85% and 40%, respectively, and capillary suction time (CST) of bioleached sludge was as low as ≈10 s. Stability and versatility of microbial communities at low pH.	[194]
	1.0‐1.5	Low‐grade CuS	*L. ferriphilum*	Copper removal efficiency was 86%. Microorganism consumed H^+^.	[156]
	2.6	Liquid‐crystal displays	*A. thiooxidans*	Indium and strontium removal efficiencies were 100% and 10%, respectively. Acidolysis was the major reaction.	[157]
	2.0	Waste printed circuits	*A. ferrooxidans*	Copper bioleaching rate reached 99%. Consumption of H^+^.	[203]
	8.0–12.5	Fly ash	*Alkalibacterium* sp. TRTYP6	Copper recovery was 52%. pH tolerant microorganism.	[158]
	7.0	e‐waste	Indigenous cyanogenic bacterial stains	Copper recovery rate enhanced up to 1.5‐5 times. Cyanide production occurs.	[159]
	1.0	Vanadium‐rich oil‐fired ash	*A. ferrooxidans*	Removal of V, Ni and Cu was obtained at 82%, 86%, and 87%, respectively. OH^−^ decreased the leaching.	[204]
	1.8‐2.0	Nickel sulfide	*A. ferrooxidans* and *S. thermosulfidooxidans*	Nickel recovery reached 50% and 80% for *A. ferrooxidans and S. thermosulfidooxidans*, respectively. Fe (III) precipitated as jarosite above pH 2.4.	[126]
	1.8	Mine tailings	*A. thiooxidans*	Enhanced leaching of Arsenic, governed by the DerjaguinLandau–Verwey–Overbeek interaction	[205]
Temperature	42 °C, 48 °C, 50 °C	Stirred tank copper sulfide	acidophilic, iron/sulfur‐oxidizing	Increase in temperature enhanced copper recovery. Optimum microbial growth temperature (50–55 °C).	[206]
	6 °C	Chalcopyrite	*Acidithiobacillus* *ferrivorans*	Polysulfide is a passivating agent, and the strain can maintain the iron‐ and sulfur‐oxidation activities at 6 °C.	[207]
	25–40 °C	Mine tailings	*A. thiooxidans*	Decreased leaching of Arsenic with an increase in temperature. Cell growth/activity decreased.	[205]
	298–313 k	Mine drainage	*L. ferriphilum*	Leaching of Zinc enhances in the range of 298–313 k	[208]
	30–50 °C	Nickel sulfide	*A. ferrooxidans and S. thermosulfidooxidans*	Nickel recovery and kinetics increased with temperature. Galvanic effect	[126]
	35 °C, 50 °C and 65 °C	Pyrite	Microbial culture	High pyrite dissolution was obtained at all three temperatures	[209]
Nutrients	NH_4_^+^, K^+^, Mg^2+^ & inorganic phosphate	Tannery sludge	*A. thiooxidans*	Inorganic phosphate enhances the oxidation of S^0^. Optimum dosage of KH_2_PO_4_ was 1.6 g L^−1^ for tannery sludge	[210]
	Sulfate ions	Pyrite	Microbial culture	Increasing sulphate concentration at all temperatures decreased the rate of sulfate dissolution	[209]
	biochar	Swine manure	Microbial community	Biochar facilitated Fe^2+^ oxidation and maintained better pH. Enhanced bioleaching was observed.	[192]
	Fe^2+^	Nickel sulfide	*A. ferrooxidans* and *S. thermosulfidooxidans*	Enhanced leaching. Supports bacterial growth and bio‐oxidation.	[126]
	Fe^2+^ & S^0^	Sludge	*A. thiooxidans* TS6 and *A. ferrooxidans* LX5	Fe^2+^ and S^0^ biooxidation rates were enhanced, which promoted the leaching rates.	[194]
	FeSO_4_.7H_2_O & S^0^	Li batteries	Thermophilic microorganisms	99.9, 99.7 and 84% of Co, Ni, and Li, respectively. Iron scrap increased Cu recovery.	[211]
	S^0^	Chalcopyrite	*A. ferrooxidans*, *L. ferriphilum* and *A. thiooxidans*	Without S^0^, Cu dissolution was 67%, however in presence of S^0^, the Cu dissolution increased to 71%. Higher S^0^ inhibited the dissolution. S^0^ promoted the growth of sulfur‐oxidizing bacteria and inhibited the iron‐oxidizing mechanism.	[212]
	Biogenic Fe^3+^	White light emitting diodes	*A. ferrooxidans*	Metal recovery rate improved by indirect bioleaching. Metals are leached by a continuous cycle of Fe^3+^ to Fe^2+^ and vice versa.	[213]
	Dissolved oxygen	Sulfide‐rich waste	“BRGM‐KCC” bacterial consortia	The oxidation rate increased with dissolved oxygen until 13 ppm due to an increase in biomass content. Microbial activity decreased when dissolved oxygen reached 17 ppm due to the formation of reactive oxygen species.	[214]
	CO_2_	Sulfitic copper concentrate	Autotrophic bacteria	CO_2_ enhanced the growth/activity of Fe‐oxidizing bacteria.	[214]
	Hole scavengers	Chalcopyrite	*A. ferrooxidans*	Photogenerated‐hole scavengers enhanced Cu leaching. Inhibited jarosite formation on metal surfaces. Enhanced Cu leaching in the combination of visible light and hole scavengers.	[215]
	Visible light and Cd^2+^	Chalcopyrite	*A. ferrooxidans*	After 28 d, Cd^2+^ showed an inhibition effect. Cu dissolution was 4.96% in visible light; however, combination of visible light and 50 mg L^−1^ of Cd^2+^ increased the dissolution to 14.70%. Cd^2+^ enhanced the attachment of microbes to the metal surface.	[216]
	UV	Low‐grade Cu tailings	*A. ferrooxidans* LD‐1	UV rays caused LD‐1 mutation. After 30 d, Cu dissolution increased by 17% with mutant bacteria. Mutant bacteria activity is better than original bacteria.	[217]
	Microwave	Nickel–laterite ores	*Bacillus subtilis*	Microwave pretreatment increased the recovery of Ni from 8% to 26% (2.3 mg Ni g^−1^ ore). Microwave preheating decreased rock strength and specific fracture energies, thereby enhancing leaching.	[218]
	Ultrasonication and acid	Spent automotive catalyst	*P. fluorescens* and *B. megaterium*	Ultrasonic pretreatment removed competing metals during leaching. After 1 d of two‐step bioleaching, *P. fluorescen*s yields 38, 44, and 91% of platinum (Pt), palladium (Pd), and rhodium (Rh), respectively. Whereas *B*. *megaterium* yields 35, 41, and 82% of Pt, Pd, and Rh, respectively. Mesophilic bacteria produced cyanide, which forms water‐soluble compounds with platinum group metals.	[219]
	Glycine	e‐waste	Cyanogenic bacteria	Glycine concentration of 2 g L^−1^. Microbes convert the glycine to HCN, thereby the optimal amount of cyanide is increased in the medium.	[159]
	Triton X‐100	Chalcopyrite	*A. ferrooxidans*	30 mg L^−1^ of surfactant increased the Cu bioleaching by 42.21% after 24 d. Surfactant induced the formation, oxidation, and elimination of S^0^ from metal surfaces.	[220]
	L‐cysteine	Low‐grade nickel and copper sulfide	Four thermophiles	80.4% Ni and 68.2% Cu recovery without L‐cysteine; while 83.7% Ni and 81.4% Cu were recovered in the presence of L‐cysteine. L‐cysteine contributed higher bacterial growth and low pH value.	[221]
	NaCl	Chalcopyrite	*Acidianus manzaensis* YN‐25	NaCl enhanced the dissolution of Cu from 2.37 to 2.67 g L^−1^. NaCl reduced the accumulation of S^0^ layers on the mineral surface.	[193]
	Pressure	Low‐grade sulfidic ore	*Leptospirillum ferriphilum*, *Sulfobacillus* sp. and *Ferrimicrobium acidiphilum*	Biological iron oxidation is enhanced by an increase in pressure from +1 to +3 bar.	[222]

**Table 5:** Influence of microbiological parameters on bioleaching of metals from inorganic wastes.

Factor	Bioleaching source	Microorganism	Experimental conditions	Findings	Reference
Microbial diversity	Copper sulfide	*A. ferrooxidans, L. ferrooxidans, Acidithiobacillus thioxidans*, and *F. acidiphilum*	pH 1.0–2.5; 45 °C; 45 d	The bacteria detected during bioleaching were *A. ferrooxidans, A. thioxidans, A. caldus, L. ferriphilum, S. acidophilus*, *and Pseudomonas aeruginosa.* Microbial richness and evenness increased at low pH.	[156]
	Copper sulfide	Fujian bacteria and Henan bacteria and mixed	0–108 h	High extraction rate by mixed bacteria. Synergistic bioleaching of sulfides by mixed bacteria at 108 h.	[223]
	Monazite	Combined neutrophilic and acidophilic bacteria	pH 2.0 30 °C	Effective bioleaching of rare‐earth elements. Synergetic interaction and generation of both sulfuric acid and organic acids.	[224]
	Low‐grade nickel and copper sulfide	Four thermophiles	pH 1.5 65 °C 16 d	Recovery of 80.4% nickel and 68.2% copper	[221]
	Pyrite	Acidophilic chemolithotrophic bacteria	pH 2.0 37 °C	Redox activity Adhesion on pyrite surface	[225]
Population density	Spent coin cells	*A. thiooxidans*	Pulp density 30 g L^−1^, 30 °C	In comparison to chemical leaching, bioleaching can enhance recovery of metals.	[118]
	Low‐grade ore	*Aspergillus* sp.	Pulp density 2% of Mn ore, pH 6.0, 37 °C	79% Mn recovery. Alteration in cellular Mn interaction.	[226]
	Chalcopyrite	Four thermophilic acidophiles	pH 1.0–2.0 40–45 °C	Addition of pyrite or sphalerite decreased the leaching process.	[227]
	CuFeS_2_	Four coculture groups and one abiotic control	30 °C 40 °C	Bacterial communities with more sulfur oxidizers showed a high Cu extraction rate.	[228]
Microbial activity	Chalcocite	*Acidithiobacillus* and *Sulfobacillus*	pH 1.8–2.0 30–35 °C	Below 700 mV of solution potential, the bacterial community showed enhanced and selective dissolution of chalcocite.	[229]
	Monazite	*Enterobacter aerogenes*	pH 3.39 48 h	Monazite dissolution was observed to decrease in the following order: biotic contact ≫ biotic noncontact ≫ spent media ≈abiotic	[224]
	Mixed oxide–sulfide copper ore	*A. ferrooxidans, S. thermosulfidooxidans, L. ferriphilum, F. thermophilum*, and *A. caldus*	Pulp density 5%; pH 2.0; 35 °C	Microbial activity and colonization are important factors for leaching efficiency. Exogenous microbial communities showed higher efficiency than indigenous microbial communities.	[228]
	Printed circuit board	Mixed culture of acidophilic bacteria	pH 2.0; Fe^2+ ^= 12 g L^−1^, 10% inoculation quantity	In a two‐step bioleaching process, 96.8% Cu and 90% of Al and Zn were recovered in 45 and 98 h, respectively. Two‐step process under optimal conditions shortened the bioleaching period from about 8 d to 45 h.	[89]

### 9.1 pH

pH plays a critical role in the growth of microorganisms. Microbial density, colonization, and metabolism are greatly influenced by a change in pH level. The pH requirement differs among a variety of microorganisms. Generally, metal solubilization occurs at low (acidophilic bacteria) and alkaline (cyanogenic organisms) pH.^[^ ^56^ ^]^ However, optimal pH conditions are essential for microbial growth and metabolism. At low pH, several heavy metals (Zn, Cu, Pb, Cr, Ni, etc.) are leached from sludge, municipal waste, and ores, amongst others. For instance, Li et al.^[^ ^117^ ^]^ recorded the highest Co recovery (47.6%) from spent LIB by *Acidithiobacillus ferrooxidans* at optimum pH 1.5. The low pH maintains the stability and versatility of the microbial community, acidolysis, jarosite formation, etc.^[^ ^126^, ^156^, ^157^ ^]^ However, an alkaline pH condition also favors the bioleaching of metals from inorganic solid wastes.^[^ ^158^, ^159^ ^]^ Remarkably, a shift in the pH from 7.0 to 9.0 resulted in enhanced (68.5%) gold recovery from PCBs by *Pseudomonas balearica* SAE1.^[^ ^98^ ^]^

### 9.2 Temperature

Temperature is an essential parameter that affects the growth and metabolism of microorganisms for metal bioleaching. Microorganisms are classified as mesophiles, moderate thermophiles, and extreme thermophiles, depending on their optimal growth temperature. The cells are active at optimal temperature but inactive at temperatures above or below their required temperatures. For instance, a higher temperature of 40 °C lessens the growth and leaching efficiency of *Aspergillus flavus*.^[^ ^160^ ^]^ The bioleaching efficacy is enhanced when carried out at the optimum temperature of the organisms.^[^ ^161^ ^]^ The majority of iron‐ and sulfur‐oxidizing bacteria grow at a temperature range of 28–30 °C.^[^ ^22^ ^]^ In addition, an increase in temperature from 30 to 35 °C resulted in maximum removal efficiencies of Li and Co from 78% and 52% to 89% and 72%, respectively, during bioleaching of metals from spent LIBs using a mixed culture of *Sulfobacillus* sp. and *Alicyclobacillus* sp. Kumar et al.^[^ ^98^ ^]^ reported a maximum recovery of 56.2% gold and 26.6% silver from waste PCBs at 30 °C in a bioleaching experiment carried out in the presence of *Pseudomonas balearica* SAE1.

### 9.3 Pulp Density

Pulp density is a measure of the quantity of solids in a solution, and it is usually expressed as a percentage. A higher liquid‐to‐solid ratio shows a lower pulp density.^[^ ^162^ ^]^ The pulp density determines the leaching efficiency of microorganisms. At higher pulp density, the toxicity level of the bioleaching medium is increased, thereby inhibiting the metabolic activity of the organisms.^[^ ^163^ ^]^ Maximum precious metals recovery was reported at optimum pulp densities of 4% (*Bacillus megaterium*), 1% (*Roseovarius tolerans* DSM 11457), and 0.5% (*Chromobacterium violaceum*).^[^ ^55^, ^102^, ^164^ ^]^ Amiri et al.^[^ ^165^ ^]^ recorded the highest recovery of Mo, Al, and W from spent hydrocracking catalysts by *Penicillium simplicissimum* at a pulp density of 3%.

### 9.4 Nutrient Composition

Culture media consist of carbon and nitrogen sources as well as nutritive salts that influence the growth and metabolic activity of microorganisms for the bioleaching of metals. In other words, the choice of suitable culture media is vital for efficient metal bioleaching.^[^ ^166^ ^]^ Synthetic nutrients, including dipotassium K_2_HPO_4_, (NH_4_)_2_SO_4_, H_3_PO_4_, and MgCl_2_, are commonly utilized to provide nutrients for microbial growth.^[^ ^56^, ^167^ ^]^ Furthermore, organic nutrient sources, including beef extract, yeast extract, and peptone, promote the growth and leaching potential of some microbes.^[^ ^53^ ^]^ Remarkably, cyanogenic bacteria (such as *Chromobacterium violaceum*) and chemolithotrophic bacteria (e.g., *Acidithiobacillus ferrooxidans* and *Thiobacillus thiooxidans*) depend on glycine, ferrous salts, and elemental sulfur to secrete HCN and sulfuric acid, respectively.^[^ ^22^, ^53^, ^168^, ^169^ ^]^ For instance, Li et al.^[^ ^117^ ^]^ reported optimum Co recovery (48.2%) from LIBs at Fe^2+^ concentration of 45 mg L^−1^ by *Acidithiobacillus ferrooxidans*. At an optimum glycine concentration of 0.5 g L^−1^, concomitant extraction of copper (13.26%) and gold (36.81%) was recorded by *Bacillus megaterium*.^[^ ^170^ ^]^

### 9.5 Dissolved Oxygen

A sufficient supply of oxygen is vital for the growth and enhanced metabolic activity of microorganisms (aerobes) for the removal of metals from solid waste materials.^[^ ^22^ ^]^ This is attained in the laboratory by aeration, shaking, or stirring. In the case of acidophilic organisms, dissolved oxygen is essential as a terminal electron acceptor for the oxidation of Fe^2+^ to Fe^3+^.^[^ ^171^ ^]^ However, less oxygen supply delays the release of sulfuric acid for metal solubilization.^[^ ^172^ ^]^ For instance, enhanced Co extraction (≈74%) from spent LIBs was recorded by *Acidithiobacillus ferrooxidans* by aeration (stirring), found to be less when compared to 52% obtained without aeration.^[^ ^173^ ^]^

### 9.6 Bioleaching Substrate

The composition of substrates, including e‐waste, fly ash, LIBs, etc., is an essential parameter that affects the bioleaching process. These inorganic waste materials consist of varying amounts of precious metals, heavy metals, and rare‐earth elements, which can be biorecovered using microorganisms.^[^ ^90^, ^109^ ^]^ Substrate particle size is an essential parameter that influences the leaching capacity of microbes. A decrease in the particle size enhances the substrate's surface area, thereby increasing the interaction of microorganisms with the inorganic waste materials and mass transfer for higher bioleaching yields.^[^ ^19^ ^]^ Particle sizes are grouped as fine (<1 mm) or coarse (1–25 mm), depending on the leaching technique.

### 9.7 Types and Physiology of Microbes

A variety of microorganisms (fungi and bacteria) are utilized for the bioleaching of metals from inorganic solid wastes. These include heterotrophic and chemolithotrophic organisms, based on their carbon and energy requirements. The microbes can be employed as wild‐type, genetically modified, pure, mixed, or consortium for metal bioleaching. The efficacy of these organisms in the removal of metals from inorganic solid waste is dependent on the inoculum volume, optimal metal tolerance, and toxicity of the waste materials.^[^ ^104^ ^]^ In addition, the viable population density of the organisms influences metal dissolution kinetics from the solid matrices.^[^ ^88^ ^]^ Increasing the microbial population is an efficient method of enhancing the effectiveness of bioleaching. A high relative abundance of *Acidithiobacillus* was vital for effective bioleaching of Cr from tannery sludge.^[^ ^174^ ^]^

## 10 Economic Impacts of Bioleaching

Since the 15th or 16th century, humans have been unaware of the causal agents responsible for the metal bioleaching process. Microbiological technology has gained attention since bacterial oxidation was used in sulfide ores to extract copper.^[^ ^175^ ^]^ Bioleaching is an ecologically beneficial technique that recycles heavy metals via natural biogeochemical cycles and is a potential method for extracting metal compounds from solid substrates or detoxifying heavy metal‐contaminated wastes.^[^ ^176^ ^]^ In the selective extraction of metals, bioleaching depends on the strains and leaching conditions. It is utilized to recover copper, nickel, cobalt, zinc, and uranium from their respective ores. Bioleaching is being employed in more than 20 industrial copper ore operations across the globe, and these activities are responsible for generating more than 20% of the world's copper each year.

Bioleaching has been explored in various applications to extract metals from oxide ores,^[^ ^177^ ^]^ metallurgical waste,^[^ ^178^ ^]^ electronic scrap,^[^ ^179^ ^]^ wastewater sludges,^[^ ^180^ ^]^ and MSW incineration fly ash.^[^ ^63^ ^]^ Rigorous work has been done on the efficiency of bioleaching of metals from primary and secondary source materials using various types of waste.^[^ ^181^ ^]^ Bioleaching is usually very simple and cheap and requires low energy, pressure, temperature, and chemical conditions. Microbial bioleaching is a cost‐effective metal recycling process from a low‐grade ore.^[^ ^182^ ^]^ The eco‐friendly, cheap, and nontoxic fungal bioleaching leads to the economic recovery of metals by reducing pollution and protecting the environment. Bioleaching could be extended to extract nanomaterials from minerals such as ilmenite, chromite, magnetite, bauxite, etc., from the natural environment.

Microorganisms play a pivotal role in the bioremediation of toxic metals and radionuclides, thus resolving many problems, such as energy and landfill space. They are important for industry and ecology because they solubilize the metals from ores. To minimize corrosion and acid deposition in the atmosphere, these organisms eliminate heavy metals from polluted industrial effluents or soils and desulfurize fossil fuels. A variety of microbes have been efficiently used for metal leaching from mineral and waste streams. Precious metals recovered by recycling of e‐waste are employed in jewelry, electronics, aviation, dentistry, and automotive industries owing to their chemical resistance and electrical conductivity.^[^ ^25^, ^56^ ^]^ In the bioleaching process, microorganisms are easily adapted to extreme living conditions.^[^ ^183^ ^]^ Thermophilic microbes can endure greater temperatures and are resistant to the metals that are being extracted. For instance, *Chromobacterium violaceum*, *Pseudomonas plecoglossicida*, and *Pseudomonas fluorescens* were used to extract Au, Ag, and Pt from electronic, jewelry, and automotive catalytic converter waste.^[^ ^41^ ^]^ Ilyas et al.^[^ ^151^ ^]^ extracted metal from electronic scrap using moderately thermophilic bacteria. The mixed consortia of microorganisms improved metal dissolution. The bioleaching organisms are thermophiles with high metal tolerance and with the potential to grow in acidic and high‐temperature environments, making the organisms very beneficial for extracting metals. The bioleaching process is enhanced by gradually adapting microbes to their surroundings and adding acidifying chemicals.^[^ ^20^ ^]^

## 11 Current Challenges Affecting Bioleaching Process

Several factors influence the bioleaching of metals from different sources. Among these, toxicity and jarosite formation are the two major hindrances influencing the bioleaching process. These limitations are discussed in detail later.

### 11.1 Toxicity

The heavy metals, nonmetals, and organic compounds in several sources, such as ores, metallurgical wastes, and e‐wastes, pose a threat to the growth and metabolism of microbes. The nonmetals in the PCBs are toxic to microbes during the bioleaching process.^[^ ^184^ ^]^ Rodrigues et al.^[^ ^185^ ^]^ found that the fluoride toxicity parameter (η) in column bioleaching of copper sulfides with fluoride‐bearing copper sulfides by *Acidithiobacillus ferrooxidans* changes with the amount of ferric ion and aluminum in the leachate. The adaptation of microorganisms to the heavy metal environment is essential for the bioleaching process. Jang and Valix^[^ ^186^ ^]^ reported that metal concentration, acid generation, and adaptation period played a dominant role in the bioleaching of heavy metals by *Acidithiobacillus thiooxidans*.

### 11.2 Jarosite

Precipitation of target metal as jarosite adversely decreases the metal recovery rates and affects the bioleaching process. Jarosites are iron–hydroxy sulphate minerals represented as M_n_(Fe^3+^)_6_(SO_4_)_4_(OH)_12_, where M could be K, NH_4_, Na, Ag, or Pb; n equals 1 and 2 for monovalent and divalent cations, respectively.^[^ ^187^ ^]^ Jarosite formation acts as a passivation layer to reduce the leaching of heavy metals.^[^ ^188^ ^]^ It acts as a clogging material on the inorganic solid waste, which influences the colonization of the microbial community and the stability of the bioprocess in the leaching of PCBs.^[^ ^189^ ^]^ However, the formation of elemental sulfur and jarosite does not affect the passivation of the arsenopyrite surface in acid mine drainage.^[^ ^190^ ^]^ Hong et al.^[^ ^191^ ^]^ reported that jarosite and elemental sulfur formation act as a hindrance in bornite bioleaching from semiconductor materials. Wei et al.^[^ ^192^ ^]^ demonstrated that vanadium leaching efficiency decreased because of both adsorption and coprecipitation with jarosite. Chang et al.^[^ ^193^ ^]^ reported that the formation and evolution of secondary minerals, such as elemental sulfur, jarosite, bornite, and chalcocite, decreased the bioleaching efficiency. The use of limonite as a seed crystal stimulates the production of goethite while inhibiting the formation of jarosite. Reintroducing a sulfur‐enriched community to ferrous and sulfur oxidizers slowed down the jarosite formation in chalcopyrite bioleaching.^[^ ^194^ ^]^ The addition of L‐cysteine inhibited jarosite formation by enhancing pyrite bio‐oxidation on the coal surface.^[^ ^195^ ^]^

### 11.3 Other Challenges

Bioleaching is a slow process that is commonly associated with low yields of desired metals from solid waste materials. This is due to the limited secretion of biolixiviant (e.g., HCN, organic acids) by microorganisms. The use of advanced and innovative technologies, including genetic engineering, bioreactors, artificial intelligence, robotics, and machine learning, is a promising approach for the commercial recovery of metals from solid waste materials. In addition, the slow kinetics of metal bioleaching can be ameliorated by the use of appropriate catalysts (metals or nonmetal ions) for enhanced catalysis and greater metal–microbe interactions for maximum recovery of metals from solid waste materials.^[^ ^56^ ^]^

## 12 Conclusions and Recommendations for Future Perspectives

Due to rapid global industrial growth and incessant use of modern technologies, there is an increase in indiscriminate disposal and accumulation of inorganic solid waste. In such a situation, implementing eco‐friendly and low‐cost bioleaching technology using microorganisms is crucial to alleviate the challenges of waste management. There are many reports involving the use of bioleaching for the synthesis of nanoparticles coupled with the recovery of metals, which makes it a highly modernized aspect for applying two processes simultaneously. To implement bioleaching at a large scale, mathematical modeling, along with computational simulations, is employed to study growth kinetics, optimize bioprocess parameters, and elucidate interactions among parameters. Mathematical modeling is an important aspect of optimizing the whole bioleaching process. The process optimization through this method, like RSM, not only depicts the optimum parameters but also reveals the possibility of microorganisms for the bioleaching process.

Bioleaching will be a key, sustainable, and prominent technology for recovering metals from inorganic waste in the future. It not only stands as an eco‐friendly and low‐cost method but also permits the possibility of mutations of microorganisms, which must be addressed due to the stress conditions during the operation. Proper disposal of microorganisms after use in bioleaching is a crucial concern for the release of mutated hazardous materials into the environment. Hence, there is a need to design appropriate biological models with complete optimized parameters. However, adequate disposal of nanoparticles is a vital challenge nowadays. In such a situation, building biological models may play a significant role. Bioleaching is used to extract metals from ores, where specific mathematical models and computational simulations are performed. So, to avoid the re‐implementation of the same models for a similar process, it is important to generate new parameter‐based databases, which will be microorganism, metal, and metal ore specific. This will save time and be a helpful tool for people working on bioleaching. Further, the bioleaching process can achieve enhanced and efficient metal recovery by adjusting the reaction parameters. Along with this, there is a need to find new approaches and visualize the behavior of interparticle and intraparticle flow. Sustainable development, environmental integrity, and habitats for living organisms can be preserved by a wise disposal of inorganic waste. However, accomplishing this target would require better waste management strategies and regulations in developed and developing nations.

## Conflict of Interest

The authors declare no conflict of interest.
